# Patient experience of head and neck treatment on a 1.5 T MR-Linac: is the ATS-lite adaptive solution tolerable?

**DOI:** 10.1016/j.tipsro.2025.100324

**Published:** 2025-07-21

**Authors:** Helen Barnes, Sophie Alexander, Shreerang Bhide, Alex Dunlop, Amit Gupta, Kevin Harrington, Trina Herbert, Kee Howe Wong, Helen McNair

**Affiliations:** a Royal Marsden NHS Foundation Trust, United Kingdom; b Institute of Cancer Research, United Kingdom

## Abstract

**Introduction:**

Head and neck cancer (HNC) treatment on the Unity MR-Linac (MRL) (Elekta AB, Stockholm, Sweden) has been developed using the novel adapt-to-shape Lite (ATS-lite) method to create clinically acceptable adaptive treatments clinician-free. Here we investigate patient experience and acceptability of this technique.

**Methods:**

Ten HNC patients treated to 65 Gy in 30 fractions with MRI-guided adaptive radiotherapy (MRIgART) within the PERMIT trial (NCT03727698), were included. Data collected comprised patient demographics, treatment time, and patient experience, using an established MRL questionnaire.

Back-up plans were created for use on the conventional linac with CT guidance, to prevent missed fractions. The frequency of use was collected and categorised to reflect the cause.

**Results:**

The median total treatment time for ATS-lite method was 39 min. The percentage of treatments under 60 mins was 98.8 %.

Questionnaire response rate was 85% and individual question response rate was 99%. Ninety-six percent of responses scored 2 or 3 on the Likert scale, a positive answer. The lowest scoring question was “I forced myself to manage the situation,” with a mean (SD) of 2.4 (0.9).

The MRL delivered 84.7 % of treatments. The back-up plan was used for 46 fractions, 7 attributed to patient tolerance (n = 2 patients).

**Conclusion:**

Average treatment times for the ATS-lite HNC MRIgART are acceptable and faster than reported ATP treatment times. Patient-reported experience was extremely positive. Use of back-up plans attributable to lack of patient tolerance was low. This technique can used with the confidence that patient experience is not negatively impacted.

## Highlights

• Investigating patient tolerance is paramount when implementing new radiotherapy techniques.
• Patient reported experience of head and neck treatment on the MR-Linac is positive.
• MRI guided adaptive radiotherapy for HN cancer does not negatively impact the patient experience.

## Introduction

Radiotherapy for patients with head and neck Cancer (HNC) on the MR-Linac (MRL) has recently been developed and put into practice using the novel adapt-to-shape Lite (ATS-lite) adaption method, developed by Gupta *et al*. (2021) [1] on the Elekta Unity system (Elekta AB, Stockholm, Sweden). This method provides a compromise between the adapt-to-position (ATP) and adapt-to-shape (ATS) workflows which have been previously defined by Winkel *et al*. (2019) [2]. Earlier work on online adaptive HNC treatment planning had shown that the ATP method does not produce plans that routinely satisfy dosimetric clinical goals [3] and the full ATS workflow requires complicated and time-consuming contouring of the anatomy, which is not practical with the patient on the couch [1]. ATS-lite has been proven to produce clinically acceptable online adapted plans in terms of clinical goal compliance without the need for a clinician to be present, and with additional benefit of reducing the need to re-set-up patients or completely replan offline should set-up errors be greater than 5 mm, as reported with ATP workflows [1]. ATS-lite also accounts for changes to the external contour of the patient such as weight loss.

Analysis of the first two patients treated using ATS-lite showed excellent pass rates of mandatory clinical goals and acceptable treatment session durations [1]. That study was limited by the small patient numbers and lack of patient-reported experience data and, therefore, the patients’ tolerance of the technique has not been fully investigated. Similarly, McDonald *et al.* (2021) [4] also investigated feasibility and efficacy of MRIgART online planning in a cohort of ten patients, but with no sampling or discussion around the patient experience. Other studies have looked at patient acceptability of MRIgART treatment across multiple body sites [5,6] and patient-reported outcomes (PRO) in HNC radiotherapy in general has been investigated [7,8]. However, little research has been conducted to date on the patient tolerance and acceptability of HNC MRIgART. This is of great importance given what we already know about elevated levels of anxiety and toxicity burden in patients with HNC who receive radiotherapy [9,10] and the potential for extended treatment times for MRIgART [2].

## Materials and methods

Ten patients were included in the study, all receiving MRI-guided adaptive radiotherapy (MRIgART) to the head and neck region under the PERMIT trial (NCT03727698) using the ATS-lite technique. Data collected as part of the trial protocol were analysed retrospectively, including patient demographics, treatment times, number of back-up plan treatments delivered and patient experience.

Patients were immobilised as per department protocol; supine and head-first in a 5-point shell HN system, with a closed face design. Internal foam ear plugs were inserted into the auditory canal and external over-ear headphones were placed over the thermoplastic shell to maintain the hospital policy for two levels of hearing protection in an MRI environment. Session and verification scans were T2 weighted 3D MR images and intrafraction imaging was carried out using a bTFE motion monitoring sequence with manual gating. Additional research imaging was carried out during the optimisation phase and included a T2 3D large field of view, T2 SPAIR, 3Dvane, DWI and MRF sequence, with each acquired weekly on a different day to minimise the impact of additional scans to the patient. Deformable propagation of the external contour from the reference plan was carried out at each fraction to account for interfraction contour change. All other contours were propagated rigidly as per the ATS-lite method described by Gupta *et al.* (2021) [1]. This is to reduce the time required for online adaptation and to free the clinician from the need to attend daily for target and organ-at-risk contouring. In-bore ventilation was maintained at level three throughout the session, the minimum required during scan acquisition according to local department procedures. This could be reduced by the radiographers when no scans were being acquired, at the patient’s request or after review of patient experience questionnaire answers. However, changes to ventilation were not recorded in this study. Communication with the patient was typically carried out at standard timepoints during each session, for example prior to scan acquisition or delivery of the radiation, however this is at staff discretion and can vary. If a patient requests more regular communication or feedback is received via the questionnaire, then the radiographers will speak to the patient more often over the intercom.

### Treatment time

Treatment times were recorded for every session. Treatment time was defined as total time from the start of the session, to beam off, to allow for direct comparison with previously published treatment times for HNC MRIgART [4].

### Back-up plan treatments

HNC is classed as Category 1 by the Royal College of Radiologists (RCR) who define this as “patients with the tumour types for which there is evidence that prolongation of treatment affects outcome…” [11]. To fulfil this and prevent gaps in the treatment schedule, under the PERMIT trial (CCR 4841), these patients had two treatment plans created at the pre-treatment phase, an MRL plan and a back-up plan. The back-up plan is a standard-of-care plan for use on a CBCT linac, which can be used in the event of MRL unavailability or if the patient is unable to tolerate the MRL treatment because of the additional time involved. It is important to determine the rationale for back-up plan use to distinguish between those attributable to patient intolerance of the MRL procedure and those due to machine unavailability. This information is recorded locally for every instance and was accessed retrospectively for analysis. The use of these plans was split into three classifications:

• scheduled back-up plan use − routine maintenance and servicing that had been planned in advance or public holidays
• unscheduled (machine unavailability) − machine breakdowns and maintenance that has not been planned in advance; and
• unscheduled (patient tolerance/side effects) − fractions where the patient felt unable to endure the MRL treatment, for example, due to anxiety or treatment-related side effects.

Frequent use of back-up plan use related to lack of patient compliance (patient tolerance/side effects) would suggest the procedure is not well tolerated.

### Patient-reported experience

Routine data collection under the PERMIT trial protocol included a patient experience questionnaire developed and validated by Barnes *et al*. (2021). A copy of the questionnaire content can be seen in supplementary material. This was completed immediately after treatment for the first three treatments and the final treatment. The data collected were post-processed in line with previous studies [5,12]*,* for simplified analysis and direct comparison with previously published patient experience data relating to a mixed cohort of patients with a range of body sites treated using MRIgART on an MRL [5]. Post-processing generated results where scores of 2 and 3 on the Likert scale always represented a favourable outcome and scores of 0 and 1 a less favourable outcome, across all questions, regardless of whether the question is positively or negatively phrased.

Patient reported experience was analysed against total treatment time using a spearman’s rank correlation coefficient. This included the total questionnaire scores, the total from all questions within the questionnaire at a single timepoint, and the two lowest scoring questions within the questionnaire.

## Results

Initial recruitment into the PERMIT trial for MRIgART HNC treatment happened over thirty months and of the ten patients originally recruited, patient number five withdrew from the PERMIT trial prior to commencing treatment on the MRL and was transferred to the main department for standard of care treatment using CT linacs. This patient was, therefore, excluded, and the eleventh patient was included to maintain the sample size of ten. All participants were male with squamous cell carcinomas requiring bilateral neck irradiation. Median (range) age was 73 (60–77) years. Staging ranged from T2N0M0 to T4aN2M0 (9 oropharynx and 1 larynx) and eight were p16 positive.

### Treatment time

The median (range) total treatment time for ATS-lite method was 39 min (22–73 min). Treatment times were compared with previously published data from a centre treating HNC on a 1.5 T MR-Linac using the ATP adaptation method, where the median (range) was reported as 46 min (31–85 min) [4]. The longest recorded treatment time recorded was 73 mins for ATS-lite and 85 mins for ATP [4]. The percentage of treatments completed in under 60 min was 99 % compared to 91 % in the previous study [4].

### Back-up plan treatments

**Fig. 1:**
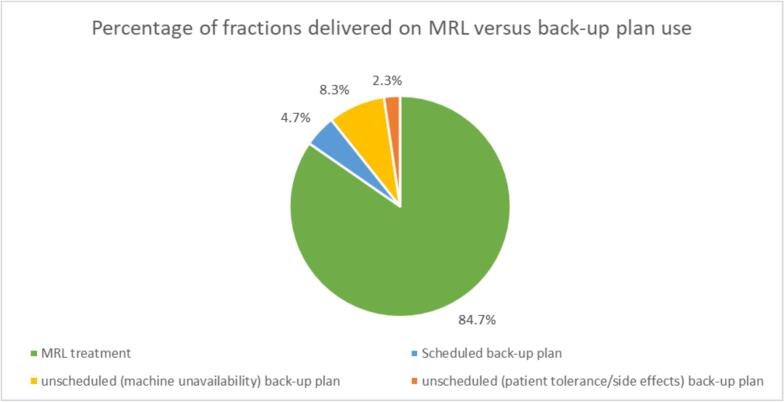
Categorised use of back-up plans.

Of the 300 fractions delivered, 254 (84.7 %) were delivered as planned on the MRL (Fig. 1). The back-up plan was used for a total of 46 fractions, 2 % of which were unscheduled related to patient tolerance or side effects. These 7 fractions were attributed to 2 different patients and occurred towards the end of their treatment courses. Patient 4 requested back-up plan use at fraction 24 due to pain and excess mucus production. This was the patient’s decision with staff agreement. Patient 1 required back-up plans for fractions 25–30 due to complications after insertion of a nasogastric feeding tube, this decision was at staff discretion.

### Patient-reported experience

**Fig. 2:**
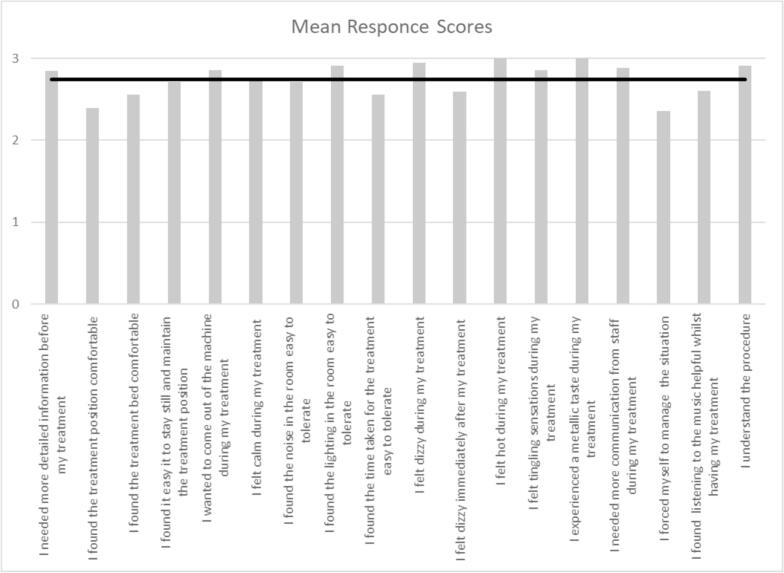
Patient experience, post-processes mean scores by question (where 3 = positive response and 0 = negative response).

The response rate to the patient-reported experience questionnaire was 85 %. The mean (SD) score across all questions (Fig. 2) was 2.7 (0.2), where the maximum possible is 3. Two questions scored the highest possible result. These were “I felt hot” and “I experienced a metallic taste during treatment,” The lowest scoring question was “I forced myself to manage the situation,” with a mean of 2.4.

Overall, 96 % of all questionnaire responses returned a positive answer (2 or 3). Comparison of overall scores and highest/lowest scoring questions with previous patient data collected across multiple countries and for multiple tumour sites can be seen in Table 1.

**Table 1:** Comparison of patient experience.

	This study	Barnes *et al*. (2021) [5]
Cohort	HNC (100 %)	Pelvic (35 %), abdominal (26 %), thoracic (10 %), HNC (10 %), other sites (19 %)
Overall Percentage favourable responses (scores 2 and 3)	96 %	84 %
Highest scoring question(s)	“I experienced a metallic taste during my treatment” & “I felt hot during my treatment”	“I experienced a metallic taste during my treatment”
Lowest scoring question(s)	“I forced myself to manage the situation” & “I found the treatment position comfortable”	“I forced myself to manage the situation”

Comparison of total questionnaire scores (all questions at a single fraction) with treatment time using a Spearman’s coefficient gave r_s_ (p) = −0.45 (0.019). The two lowest scoring questions were also analysed for correlation with treatment time. “I forced myself to manage the situation” r_s_ (p) = −0.43 (0.025) and “I found the treatment position comfortable” r_s_ (p) = −0.37 (0.059).

## Discussion

### Treatment time

While the ATS-lite adaptive treatment time is expected to be longer than non-adaptive treatments on a standard CBCT linac [1], it is important to compare the timings of the ATS-lite workflow against previously published timing data for other online adaptive MRL workflows at a centre also using the Elekta Unity [4]. This comparison shows That ATS-lite is faster both the median treatment time and in the worst-case scenario (longest session time) than ATP workflows. This is likely due to the fact that unlike ATP, ATS-lite does not routinely require the patient to be re set up, and ATS-lite always achieves all clinical goals [1] meaning no time is spend with decision making regarding plan quality, which can be the case with ATP workflows where clinical goals are less likely to be achieved. The ATS-lite adaptive method can, therefore, be considered an attractive alternative to ATP for treatment of HNC with regards to treatment time and on-line adaptive planning.

### Back-up plans

The unscheduled use of back-up plans related to patient tolerance or side effects is what reflects the acceptability of the treatment to patients. This non-compliance rate was low (2 %) showing that patients tolerate the 30-fraction treatment course on the MRLinac well, especially when compared to historical treatment compliance rates of 48.9 % for HNC radiotherapy [13]. The successful completion of all 300 fractions of radiotherapy is encouraging when breaks in treatment are known to have a detrimental impact on patient outcomes [14] and provides confidence that patients can tolerate HNC MRIgART with the knowledge that the treatment regime is likely to be completed in a standard and optimal time. Though these results are encouraging, it is not suggested to stop creating back-up plans for RCR Category 1 patients. Centres with multiple MRL units can transfer patients to another MRL to maintain consistent MRIgART delivery, whilst those centres with one MRL may utilise the back-up CBCT linac plan to fulfil uninterrupted treatment regimes.

### Patient-reported experience

Overall high mean scores and percentage of positive responses represents a good outcome for patient experience in this study. The patient experience questionnaire is designed to enquire about themes which are likely to affect the patient experience in an MRIgART environment [5]. These results show that despite the potential for negative experiences caused by the treatment modality and environment e.g. dizziness or noise, patients did not routinely feel negatively about the situation or treatment experience.

The lowest scoring question was “I forced myself to manage the situation” and though the mean score of 2.4 is still in the positive range, it tells us that some patients did have to force themselves to manage the treatment session and were subjected to increased levels of anxiety. This is in keeping with previous findings that an MRI environment has the potential to increase patients’ anxiety and discomfort, although these can range from apprehension and claustrophobia to panic attacks, [15] the most extreme of which were not seen in this study. Other low scoring (more negative responses) questions included: “I found the treatment position comfortable;” “I found the treatment bed comfortable;” “I felt dizzy immediately after my treatment;” and “I found the time taken for the treatment easy to tolerate.” These reinforce previous statements that MRI and radiotherapy environments have the potential to subject patients to both physical and environment discomfort [12,16].

A comparison of patient experience questionnaire results from a previously published paper [5] can be seen in Table 1 of the results section. This study also collected patient experience data from patients treated on 1.5 T MRLs across 5 different centres, using the same validated patient experience questionnaire. The study by Barnes *et al*. (2021) [5] included 170 patients with a range of tumour sites including pelvic region, abdominal (pancreas and liver), thoracic (partial breast), head and neck and other sites, including oligometastatic disease. Direct comparison shows more favourable results for the HNC patients in this study, suggesting that this treatment was better tolerated than the patients in the previous study who underwent treatments for tumour sites other than HNC which are expected to be more easily tolerated. The highest and lowest scoring questions were the same across both studies, which tells us that the themes that cause patients the most and least discomfort do not vary between different tumour sites on the MRL.

The high mean score (positive responses) for the question “I needed more communication from staff during my treatment”, where most answers were “not at all”, tells us that the radiation therapists (RTT) typically communicated at appropriate intervals during the treatment session and that lack of communication was not a source of increased discomfort for the patients. This is a similar finding to a previous MRL PRO study [6] which concluded that “Patients report that regular audio communication with radiation therapists during treatment (informing them on progress and reassuring them) was very helpful.” However, Tetar [6] found that 60 % of patients treated on a 0.35 T MRL reported “disturbing noise” as a complaint despite use of headphones. In contrast, this study had a mean score of 2.7 (out of 3) for “I found the noise in the room easy to tolerate”. This may be due to the differing MRL systems and / or sequences used, or the routine use of two levels of hearing protection at this centre: foam earplugs and headphones. The negative correlation between the total questionnaire scores with treatment time tells us that questionnaires scored lower as treatment times increased and though the correlation is weak, the p value of less than 0.05 confirms that the trend is statistically significant. Overall, the patients experience is less positive on days where the treatment takes longer to deliver. This gives us a clear area for improvement in the future, to reduce treatment times to improve the patient experience further. The two lowest scoring questions (“I forced myself to manage the situation” and “I found the treatment position comfortable”) were also compared against treatment time. While both showed a weak negative correlation with treatment time, only “I forced myself to manage the situation” produced a significant result. Patients are therefore more likely to feel as if they must force themselves to manage the treatment when delivery takes longer. The two highest scoring questions “I felt hot during my treatment” and “I experienced a metallic taste during my treatment” were not analysed for correlation with treatment time as all responses were the same (“not at all”) and therefore the scores could not be ranked.

## Conclusions

We have shown that average treatment times for the ATS-lite HNC MRIgART are acceptable to patients and faster than treatment times for the ATP online adaption method used at other centres.

Patient-reported experience was positive and showed improvement over experiences of a mixed cohort of patients also treated with MRIgART. The key themes most likely to cause anxiety with MRIgART, such as environmental and physical discomfort, remain consistent and RTTs are still shown to be key in reducing anxiety for patients through their care and communication.

Use of back-up plans relating to patient tolerance issues was low, showing that patients manage HNC MRIgART well.

This treatment method can continue to be used with the confidence that not only is the treatment effective, but that patient experience is not being negatively impacted.

## Informed patient consent

The author(s) confirm that written informed consent has been obtained from the involved patient(s) or if appropriate from the parent, guardian, power of attorney of the involved patient(s); and, they have given approval for this information to be published in this case report (series).

## Funding

This report is independent research supported by the National Institute for Health Research and Health Education England (HEE/NIHR ICA Programme Senior Clinical Lectureship, Dr Helen McNair, ICA-SCL-2018-04-ST2-002). We also acknowledge NHS funding to the NIHR Biomedical Research Centre at The Royal Marsden and The Institute of Cancer Research.

The Institute of Cancer Research is supported by Cancer Research UK Programme Grants (C33589/A19727) and the CRUK ART-NET Network Accelerator Award (A21993); MRC Grant MR/M009068/.

## Declaration of competing interest

The authors declare the following financial interests/personal relationships which may be considered as potential competing interests: This report is independent research supported by the National Institute for Health Research and Health Education England (HEE/NIHR ICA Programme Senior Clinical Lectureship, Dr Helen McNair, ICA-SCL-2018-04-ST2-002). We also acknowledge NHS funding to the NIHR Biomedical Research Centre at The Royal Marsden and The Institute of Cancer Research.

The Institute of Cancer Research is supported by Cancer Research UK Programme Grants (C33589/A19727) and the CRUK ART-NET Network Accelerator Award (A21993); MRC Grant MR/M009068/.

The views expressed in this publication are those of the author(s) and not necessarily those of the NHS, the National Institute for Health Research or the Department of Health and Social Care.

ICR/RMH is a member of the Elekta MR-Linac Research Consortium.

.

## References

[1] Gupta A., Dunlop A., Mitchell A., McQuaid D., Nill S., Barnes H. (2021). Online adaptive radiotherapy for head and neck cancers on the MR linear accelerator: introducing a novel modified adapt-to-shape approach. Clin Transl Radiat Oncol.

[2] Dennis Winkel, Gijsbert H. Bol, Petra S. Kroon, Bram van Asselen, Sara S. Hackett, Anita M. Werensteijn-Honingh, Martijn P.W. Intven, Wietse S.C. Eppinga, Rob H.N. Tijssen, Linda G.W. Kerkmeijer, Hans C.J. de Boer, Stella Mook, Gert J. Meijer, Jochem Hes, Mirjam Willemsen-Bosman, Eline N. de Groot-van Breugel, Ina M. Jürgenliemk-Schulz, Bas W. Raaymakers. Adaptive radiotherapy: The Elekta Unity MR-linac concept. Clin Translat Radiat Oncol, 18, 2019, 54–59, ISSN 2405-6308. https://doi.org/10.1016/j.ctro.2019.04.001. 10.1016/j.ctro.2019.04.001 PMC6630157 31341976

[3] A.M.A. Dunlop, I. Hanson, H. Barnes, D. McQuaid, B. Ng-Cheng-Hin. MRgRT workflow development and recommendations for H&N treatment using the Elekta Unity MRLinac MR in Radiotherapy Symposium (Abstract) 2021.

[4] McDonald BA, Vedam S, Yang J, Wang J, Castillo P, Lee B, Sobremonte A, Ahmed S, Ding Y, Mohamed ASR, Balter P, Hughes N, Thorwarth D, Nachbar M, Philippens MEP, Terhaard CHJ, Zips D, Böke S, Awan MJ, Christodouleas J, Fuller CD; MR-Linac Consortium Head and Neck Tumor Site Group. Initial Feasibility and Clinical Implementation of Daily MR-Guided Adaptive Head and Neck Cancer Radiation Therapy on a 1.5T MR-Linac System: Prospective R-IDEAL 2a/2b Systematic Clinical Evaluation of Technical Innovation. Int J Radiat Oncol Biol Phys. 2021 Apr 1;109(5):1606-1618. doi: 10.1016/j.ijrobp.2020.12.015. Epub 2020 Dec 16. PMID: 33340604; PMCID: PMC7965360. 10.1016/j.ijrobp.2020.12.015 PMC7965360 33340604

[5] Barnes H, Alexander S, Bower L, Ehlers J, Gani C, Herbert T, Lawes R, Møller PK, Morgan T, Nowee ME, Smith G, van Triest B, Tyagi N, Whiteside L, McNair H. Development and results of a patient-reported treatment experience questionnaire on a 1.5 T MR-Linac. Clin Transl Radiat Oncol. 2021 Jun 29;30:31-37. doi: 10.1016/j.ctro.2021.06.003. PMID: 34307911; PMCID: PMC8283148. 10.1016/j.ctro.2021.06.003 PMC8283148 34307911

[6] Tetar S., Bruynzeel A., Bakker R. (2018). Patient-reported outcome measurements on the tolerance of magnetic resonance imaging-guided radiation therapy. Cureus.

[7] Steen-Olsen E.B., Stormoen D.R., Kristensen C.A., Vogelius I.R., Holländer-Mieritz C., Pappot H. (2022). Patient-reported outcome during radiotherapy for head and neck cancer: the use of different PRO questionnaires. Eur Arch Otorhinolaryngol.

[8] McQuestion M., Fitch M. (2016). Patients' experience of receiving radiation treatment for head and neck cancer: before, during and after treatment. Can Oncol Nurs J.

[9] Macedo DR, Neris RR, Anjos ACY, et al. Radiotherapy experiences from the perspective of head and neck cancer patients: integrative literature review. Rev Fund Care Online. 2019; 11(3):785-791. DOI: DOI: 10.9789/2175-5361.2019.v11i3.785-791.

[10] Sarah W., Harvey Q., Colleen S., Adam Y., Lisa B., Nabhya H. (2021). Patient-reported outcomes-guided adaptive radiation therapy for head and neck cancer. Front Oncol.

[11] The Royal College of Radiologists (RCR). The timely delivery of radical radiotherapy: guidelines for the management of unscheduled treatment interruptions, fourth edition. London: The Royal College of Radiologists, 2019.

[12] Olausson K., Holst Hansson A., Zackrisson B., Edvardsson D., Ostlund U., Nyholm T. (2017). Development and psychometric testing of an instrument to measure the patient’s experience of external radiotherapy: the Radiotherapy Experience Questionnaire (RTEQ). Tech Innov Patient support. Radiat Oncol.

[13] O'Connor, Paul. The impact of missed fractions in head and neck radiotherapy and how they can be minimised. Radiography, Volume 19, Issue 4, 343 – 346. https://doi.org/10.1016/j.radi.2013.07.006.

[14] Michael Xiang, Michael Gensheimer, Erqi Pollom, F. Christopher Holsinger, A. Dimitrios Colevas, Quynh-Thu Le, Beth Beadle. Treatment breaks during definitive head/neck radiotherapy: survival impact and predisposing factors. Int J Radiat Oncol Biol Phys, 108 (2), Supplement, 2020, Page E39, ISSN 0360-3016. https://doi.org/10.1016/j.ijrobp.2020.02.558.

[15] Meléndez J.C., McCrank E. (1993). Anxiety-related reactions associated with magnetic resonance imaging examinations. J Am Med Assoc.

[16] Ahlander B.M., Årestedt K., Engvall J., Maret E., Ericsson E. (2016). Development and validation of a questionnaire evaluating patient anxiety during magnetic resonance imaging: the magnetic resonance imaging-anxiety questionnaire (MRI-AQ). J Adv Nurs.

