# Supplementary figures and images for: Patient experience of head and neck treatment on a 1.5 T MR-Linac: is the ATS-lite adaptive solution tolerable?
Source: Tech Innov Patient Support Radiat Oncol. 2025 Jul 21;35:100324. doi: 10.1016/j.tipsro.2025.100324 (PMC12312102; doi:10.1016/j.tipsro.2025.100324)

**Supplementary material**


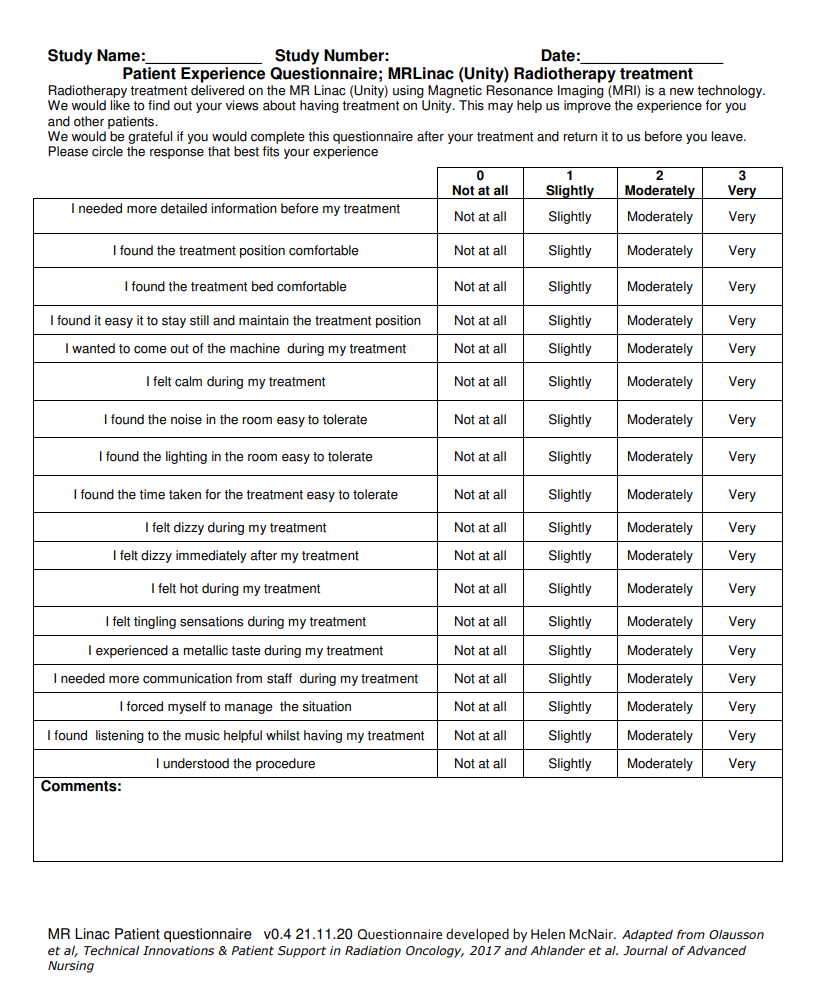
Questionnaire developed, validated and published by Barnes *et al*. (2021) [5].

Supplement: Supplementary Data 1 [file mmc1.docx]
